# Effects of Different Zinc Modulations in Glass Ionomer Cements on Multi-Species Biofilm Formation and Human Tooth Demineralization: An In Vitro Study

**DOI:** 10.3390/antibiotics15050489

**Published:** 2026-05-12

**Authors:** İpek Ören Bozyer, Khairul Matin, Tijen Pamir, Sema Belli, Yasushi Shimada

**Affiliations:** 1 Department of Restorative Dentistry, Faculty of Dentistry, Ege University, İzmir 35040, Türkiye; ipek.oren@ege.edu.tr; 2 Department of Cariology and Operative Dentistry, Graduate School of Medical and Dental Sciences, Institute of Science Tokyo (Formerly; Tokyo Medical and Dental University), 1-5-45 Yushima, Bunkyo-ku, Tokyo 113-8549, Japan; matope@tmd.ac.jp (K.M.); Shimada.ope@tmd.ac.jp (Y.S.); 3 Department of Oral Medicine and Stomatology, School of Dental Medicine, Tsurumi University, Tsurumi, Yokohama 230-8501, Japan; 4 Medoc International, Tokyo 102-0094, Japan; 5 Department of Endodontics, Faculty of Dentistry, Selçuk University, Konya 42130, Türkiye; sbelli@selcuk.edu.tr

**Keywords:** dental materials, glass ionomer cements, zinc, biofilms, demineralization

## Abstract

**Background:** Biofilm formation and associated tooth demineralization are key factors influencing the clinical performance of dental materials. **Methods:** This study compared the antibiofilm and demineralization preventive effects of two zinc-modified glass ionomer cements (Zn-GICs) with a conventional GIC. Disk-shaped specimens of Caredyne Restore (CR), ChemFil Rock (CFR), and Ketac Molar (KM) (*n* = 6) were evaluated in a multi-species biofilm model using an oral biofilm reactor. Early biofilm formation was analyzed by scanning electron microscopy (after 2 h and 4 h), bacterial accumulation and water-insoluble glucan (WIG) production were quantified (after 12 h). For demineralization assessment, restored human enamel and dentin specimens (*n* = 6) including an additional resin-based control group (Dura Seal, DS) were subjected to a 14-day biofilm challenge and lesion depth was measured using swept-source optical coherence tomography and confocal microscopy. **Results:** CR showed significantly lower bacterial accumulation and WIG production than the other materials (*p* < 0.05). CFR demonstrated lower bacterial levels than KM (*p* < 0.05), whereas no significant differences were observed between CFR and KM in WIG production (*p* > 0.05). CR produced the shallowest enamel and dentin lesions, whereas DS exhibited the deepest (*p* < 0.05); however, no statistically significant differences were observed between CFR and KM in lesion depth (*p* > 0.05). **Conclusions:** CR demonstrated superior biofilm suppression and reduced demineralization, whereas CFR showed limited differences compared with the conventional GIC.

## 1. Introduction

Dental caries involve a biofilm-mediated demineralization process in which acidogenic microorganisms metabolize dietary carbohydrates, resulting in the formation of localized acidic microenvironments on the tooth surface [1]. When mineral loss progresses to cavitation, restorative intervention becomes necessary. Therefore, restorative materials are expected not only to replace lost tooth structure but also to resist biofilm accumulation and limit acid mediated mineral dissolution at the tooth-material interface [2]. In this context, glass ionomer cements (GICs), originally developed by Wilson and Kent, continue to attract considerable interest as restorative materials due to their acid-base setting reaction, ion-releasing glass phase, and intrinsic ability to chemically adhere to dental hard tissues [3].

Over the years, multiple strategies have been explored to enhance the performance of GICs, including resin modification, nanoscale filler incorporation, the addition of antimicrobial agents (e.g., quaternary ammonium salts and chlorhexidine), and metallic reinforcement (e.g., silver and gold) [4,5]. Another modification is the incorporation of zinc (Zn). In this context, the recently introduced BioUnion™ filler represents a zinc-containing bio-functional glass powder composed of silicon dioxide (SiO_2_), zinc oxide (ZnO), calcium oxide (CaO), and fluoride (F) [6]. The incorporation of zinc into glass ionomer cements has been shown to inhibit Streptococcus Mutans (*S. mutans*) [7]. In addition, zinc has been shown to alter bacterial cell membrane permeability, thereby affecting membrane potential and modulating microbial acid production. Owing to these properties, zinc is regarded as an antiplaque agent [8,9]. Accordingly, incorporation of zinc into restorative formulations is anticipated to enhance antimicrobial performance.

Given that the clinical relevance of antimicrobial modifications depends on material performance against structured biofilms, in vitro evaluation typically relies on biofilm model systems that mimic intraoral biofilm development. The oral biofilm reactor used in the present study is an in vitro model developed to simulate the oral environment and remains widely used in biofilm research [10]. *Streptococcus mutans* (*S. mutans*) is the most widely studied species in biofilm models owing to its key role in the pathogenesis of cariogenic biofilms. *S. mutans* synthesizes water-insoluble glucans from sucrose through glucosyltransferase (GTF) mediated pathways. These glucans enhance bacterial adhesion to tooth surfaces and significantly contribute to the structural integrity of the biofilm matrix [11]. *Streptococcus sobrinus* (*S. sobrinus*) accelerates the cariogenic process owing to its high acidogenic potential and invasive characteristics [12]. *Streptococcus mitis* (*S. mitis*), as an early colonizer, contributes to biofilm architecture through its coaggregation capacity and is particularly associated with the development of root caries [13]. *Streptococcus gordonii* (*S. gordonii*) is a commensal early colonizer that mediates surface adhesion via specific adhesins [14]. *Lactobacillus casei* (*L. casei*), characterized by its acid tolerance and active carbohydrate metabolism, contributes to caries progression within the deeper layers of the biofilm [15]. In this study, the bacterial species were selected based on their functional roles within the biofilm ecosystem. Collectively, these microorganisms constitute a representative cariogenic biofilm community, enabling clinically relevant evaluation of the effects of restorative materials on biofilm dynamics and tooth demineralization.

Despite the growing interest in zinc-modified glass ionomer cements, the scope of available literature remains limited. Many studies have relied on single-species biofilm models, an approach that does not adequately capture the dynamic and polymicrobial nature of cariogenic biofilm development under in vivo conditions [16]. Moreover, existing investigations have frequently evaluated only a single zinc-containing formulation, and direct comparisons of materials with distinct zinc incorporation strategies within the same experimental design appear to be lacking in the current literature. Therefore, the present study aimed to comprehensively evaluate and compare the antibiofilm performance and resistance to biofilm-mediated enamel and dentin demineralization of two zinc-containing GICs with distinct zinc incorporation strategies Caredyne Restore (CR) and ChemFil Rock (CFR) against a conventional GIC (Ketac Molar, KM). Dura Seal (DS), a resin-based material lacking the ion-releasing capacity characteristic of GIC formulations, was included in the demineralization component of the study as a control, thus serving as a reference point for evaluating the biological benefits conferred by the ionic activity of GIC-based materials.

The null hypotheses were formulated as follows: (1) the tested GIC materials would not differ significantly in bacterial accumulation, water insoluble glucan production, or early-stage biofilm formation; and (2) biofilm mediated enamel and dentin lesion depth would not differ significantly among the tested GICs and the resin-based control.

## 2. Results

### 2.1. Quantitative Assessment of Biofilm Formation

**Figure 1:**
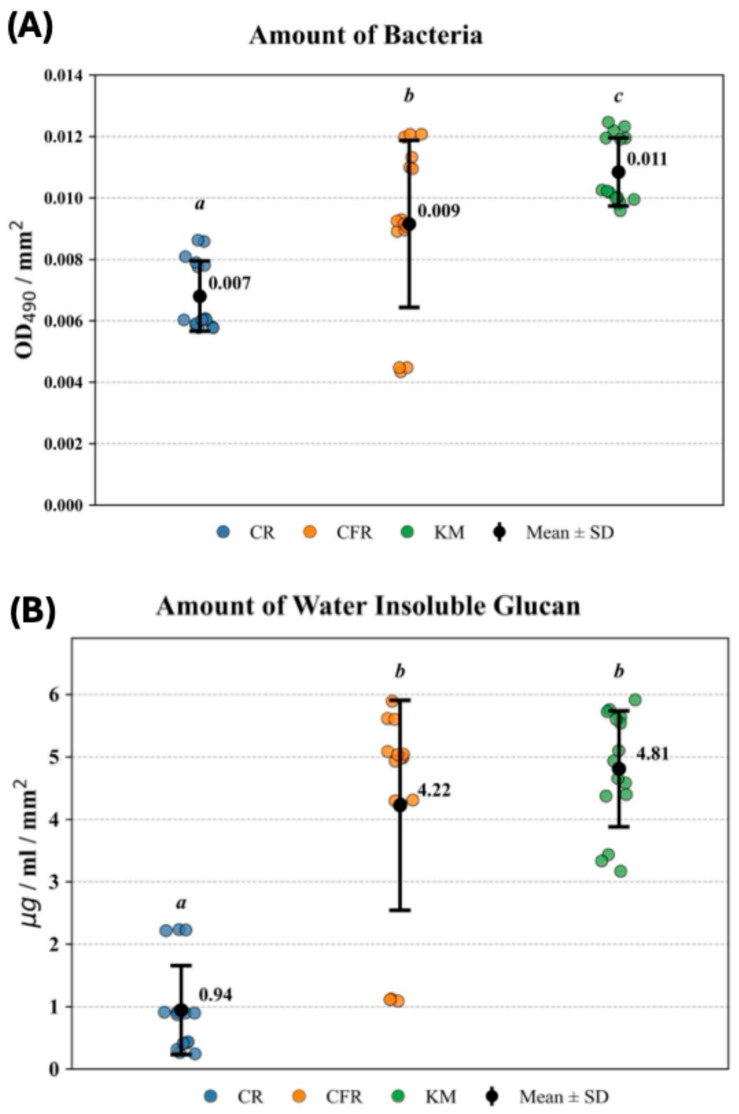
Bacterial accumulation (**A**) and water-insoluble glucan (**B**) levels on the surfaces of tested materials (CR, Caredyne Restore; CFR, ChemFil Rock and KM, Ketac Molar). Each colored data point represents an individual specimen; black circles indicate mean values with error bars representing standard deviation (SD). Statistical analysis was performed using one-way ANOVA followed by Tukey’s post hoc test. Different lowercase (*a*, *b*, *c*) letters reflect significant differences among groups (*p* < 0.05), whereas groups sharing the same letter do not differ significantly (*p* > 0.05).

One-way ANOVA revealed a significant effect of material type on bacterial accumulation (*p* = 1.59 × 10^−6^). Tukey’s HSD post hoc test showed significant differences between CR and CFR (*p* = 0.0028), CR and KM (*p* = 9.00 × 10^−7^), and CFR and KM (*p* = 0.038) (Figure 1A). A comparable pattern was observed for water-insoluble glucan accumulation, with one-way ANOVA again demonstrating a significant effect of material type (*p* = 2.18 × 10^−11^). Tukey’s HSD post hoc test identified significant differences between CR and CFR (*p* = 6.12 × 10^−9^) and CR and KM (*p* = 8.08 × 10^−11^), while no significant difference was observed between CFR and KM (*p* = 0.371) (Figure 1B).

### 2.2. Early-Stage Biofilms on Materials; Scanning Electron Microscopy (2 and 4 h)

**Figure 2:**
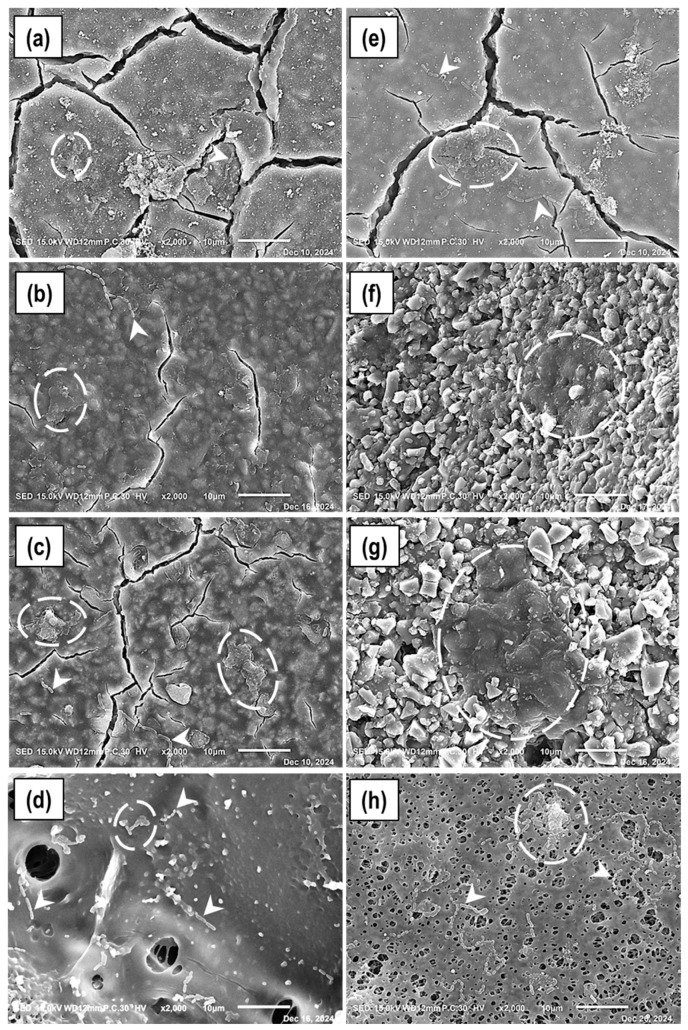
SEM images of biofilm formed on material surfaces after 2 h and 4 h. Panels (**a**–**d**): 2 h; panels (**e**–**h**): 4 h. Caredyne Restore (CR) (**a**,**e**); ChemFil Rock (CFR) (**b**,**f**); Ketac Molar (KM) (**c**,**g**); Dura Seal (DS) (**d**,**h**). White arrows indicate newly adhered bacteria (slightly elongated bacteria are lactobacilli and others are streptococci). Dotted outlines indicate forming early biofilm clusters (cemented with extracellular polysaccharides; EPS (apparently WIG); growing with the increase in time; smaller in 2 h and larger in 4 h. Scale bars = 10 μm (original magnification ×2k).

Representative SEM (JSM-5400; JEOL, Tokyo, Japan) images are shown below (Figure 2). Adhesion of bacteria was observed with a few microcolonies of early stage-biofilms on the surface of CR after 2 h (Figure 2a). At 4 h, fresh bacteria were observed to adhere on to the surface and enlarging biofilms could also be detected (Figure 2e). In addition, from the images and inspecting more surface areas by SEM it was understood that the material surface was not affected differently by the acid secreted as a result of biofilm formation for 2 and 4 h, the material surface appeared similar at both time periods. While examining the surfaces of CFR, it was observed that the bacterial adhesion and early biofilm formation were occurring simultaneously in 2 h (Figure 2b), while remarkably large and relatively condensed biofilm (with darker EPS) were formed by 4 h (Figure 2f). At 4 h, the material surface exhibited a more irregular morphology compared to 2 h. In contrast to the above two GICs, the microcolony size on KM appeared to be remarkably larger at 2 h (Figure 2c) and a more compact biofilm appearance was observed at 4 h (Figure 2g), in which EPS almost completely embedded the multi-species bacteria. At the end of the 2 h, the surface of KM appeared relatively smooth, whereas by 4 h, it exhibited a more irregular morphology. The control material (DS) surface showed scattered bacterial adhesion, but biofilm clusters were smaller in size compared to those on CFR and KM both at 2 h and 4 h (Figure 2d,h).

### 2.3. Quantitative Analysis of Enamel and Dentin Enamel and Dentin Lesion Depth Following Biofilm Challenge

**Figure 3:**
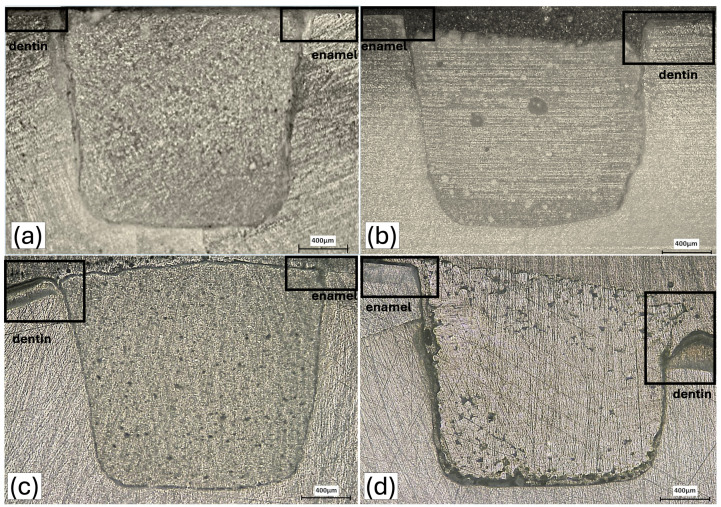
LSCM images of midline sections of the specimens following biofilm-mediated demineralization: (**a**) Caredyne Restore, (**b**) ChemFil Rock, (**c**) Ketac Molar, and (**d**) Dura Seal. Images were acquired at 5× magnification. Sections were obtained through the center of the restorations after a 14-day biofilm challenge. White rectangles indicate areas of demineralization at the enamel and dentin margins.

Both in the SS-OCT (IVS-2000, Santec, Komaki, Japan) and LSCM (VK8510, Keyence Corporation, Tokyo, Japan) images, demineralization of enamel and dentine could be detected, lesions were clearly visible in LSCM images in all samples (Figure 3). The lesions were less deep in CR and CFR compared other control materials for dentin especially.

#### 2.3.1. SS-OCT

**Figure 4:**
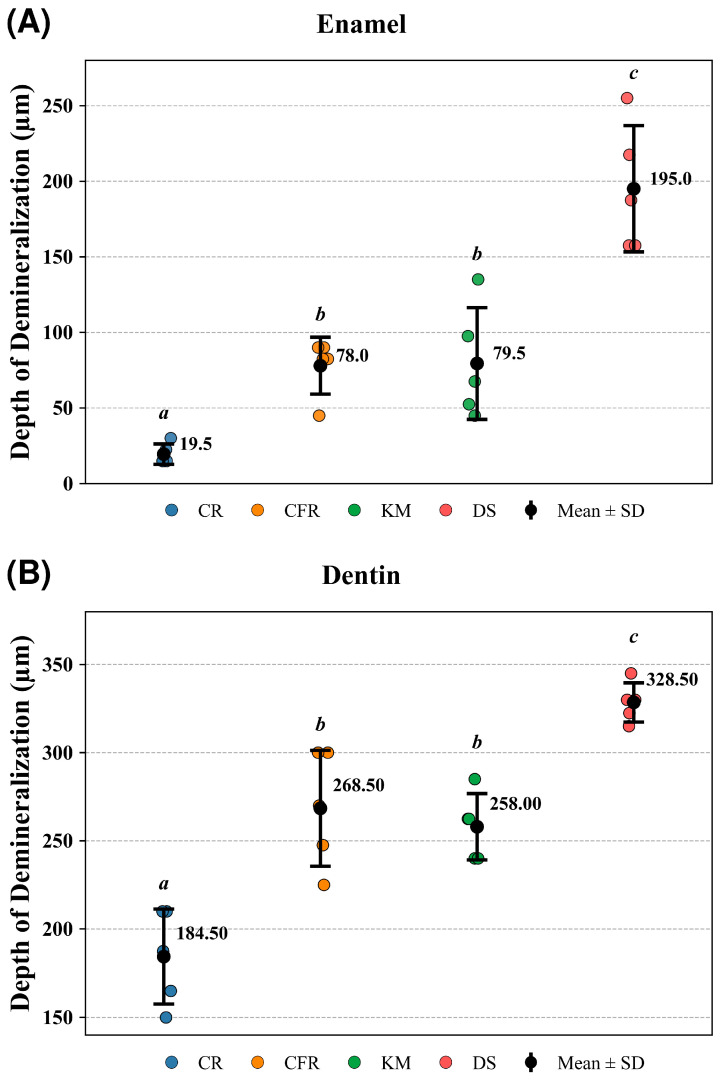
Lesion depth measured by SS-OCT in (**A**) enamel and (**B**) dentin (μm) adjacent to the tested materials (CR, Caredyne Restore; CFR, ChemFil Rock; KM, Ketac Molar; DS, Dura Seal). Each colored data point represents an individual specimen; black circles indicate mean values with error bars representing standard deviation (SD). Statistical analysis was performed using one-way ANOVA followed by Tukey’s post hoc test. Different lowercase (*a*, *b*, *c*) letters reflect significant differences among groups (*p* < 0.05), whereas groups sharing the same letter do not differ significantly (*p* > 0.05).

Based on SS-OCT image analysis (Figure 4), one-way ANOVA demonstrated that material type significantly affected lesion depth in both enamel (*p* = 7.02 × 10^−7^) and dentin (*p* = 7.27 × 10^−7^). In enamel, Tukey’s HSD post hoc test revealed that CR exhibited significantly lower lesion depths than CFR (*p* = 3.01 × 10^−2^), KM (*p* = 2.57 × 10^−2^), and DS (*p* = 3.75 × 10^−7^) while DS showed significantly greater lesion depths than CFR (*p* = 6.29 × 10^−5^) and KM (*p* = 7.29 × 10^−5^). No significant difference was observed between CFR and KM in enamel (*p* = 0.99). A comparable pattern was observed in dentin, where CR demonstrated significantly lower lesion depths than CFR (*p* = 2.26 × 10^−4^), KM (*p* = 8.83 × 10^−4^), and DS (*p* = 2.92 × 10^−7^), and DS exhibited significantly greater values than CFR (*p* = 5.39 × 10^−3^) and KM (*p* = 1.32 × 10^−3^), with no significant difference between CFR and KM (*p* = 0.9).

#### 2.3.2. LSCM

**Figure 5:**
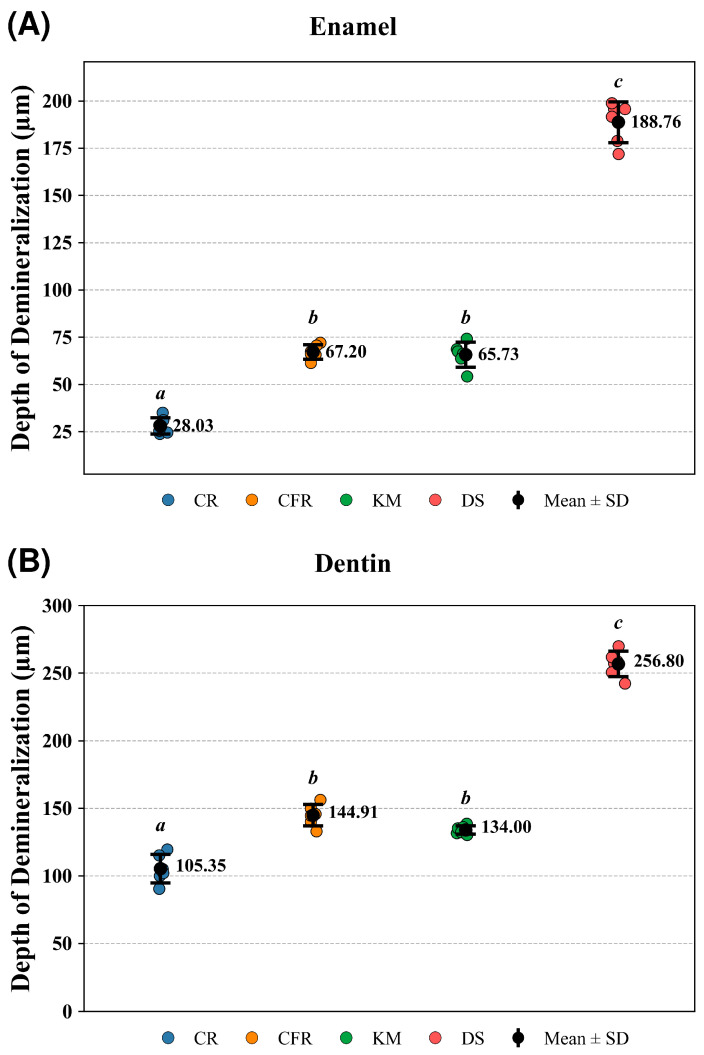
Lesion depth measured by LSCM in (**A**) enamel and (**B**) dentin (μm) adjacent to the tested materials (CR, Caredyne Restore; CFR, ChemFil Rock; KM, Ketac Molar; DS, Dura Seal). Each colored data point represents an individual specimen; black circles indicate mean values with error bars representing standard deviation (SD). Statistical analysis was performed using one-way ANOVA followed by Tukey’s post hoc test. Different lowercase (*a*, *b*, *c*) letters reflect significant differences among groups (*p* < 0.05), whereas groups sharing the same letter do not differ significantly (*p* > 0.05).

Based on LSCM image analysis (Figure 5), one-way ANOVA demonstrated that material type significantly affected lesion depth in both enamel (*p* = 9.11 × 10^−20^) and dentin (*p* = 7.37 × 10^−18^). In enamel, Tukey’s HSD post hoc test revealed that CR exhibited significantly lower lesion depths than CFR (*p* = 2.93 × 10^−8^), KM (*p* = 5.49 × 10^−8^), and DS (*p* = 2.33 × 10^−15^), while DS showed significantly greater lesion depths than CFR (*p* = 2.33 × 10^−15^) and KM (*p* = 2.33 × 10^−15^). No significant difference was observed between CFR and KM in enamel (*p* = 0.983). Similarly, in dentin, CR demonstrated significantly lower lesion depths than CFR (*p* = 4.01 × 10^−7^), KM (*p* = 4.15 × 10^−5^), and DS (*p* = 2.33 × 10^−15^), and DS exhibited significantly greater values than CFR (*p* = 5.44 × 10^−15^) and KM (*p* = 2.89 × 10^−15^), with no significant difference between CFR and KM (*p* = 0.137).

## 3. Discussion

The present study evaluated the antibiofilm and demineralization preventive performance of two zinc-containing glass ionomer cements (Zn-GICs) using a multi-species cariogenic biofilm model designed to simulate clinically relevant conditions. The findings demonstrated distinct material dependent differences in both biofilm suppression and resistance to biofilm-mediated mineral loss.

### 3.1. Antibiofilm Performance and Underlying Mechanisms

Significant differences were observed in bacterial adhesion, biofilm accumulation, and water-insoluble glucan (WIG) production. Although CFR and KM differed significantly in bacterial accumulation, this difference was not reflected in WIG levels, indicating that reductions in bacterial load do not necessarily correspond to decreased extracellular matrix synthesis. Among the tested materials, CR exhibited the lowest levels of bacterial adhesion and glucan production, consistent with a greater capacity to interfere with early biofilm establishment and matrix maturation.

Previous investigations comparing CR with conventional GICs have reported reduced bacterial accumulation and a thinner extracellular polymeric substance (EPS) layer on CR surfaces, effects attributed to the synergistic release of Zn and fluoride (F) ions [17,18]. In solution-based systems, Zn and F, either alone or in combination, have been shown to inhibit Streptococcus mutans biofilm formation by disrupting insoluble glucan synthesis [19]. Similarly, Zn-F glass nanoparticles have demonstrated inhibitory effects on Streptococcus mutans and Actinomyces naeslundii biofilm formation [20].

The present findings are consistent with these reports and suggest that the superior antibiofilm performance of CR is closely linked to its ion-release behavior. Our independent analysis demonstrated significantly higher Zn release from CR under acidic conditions than from CFR [21], supporting the enhanced biofilm inhibition observed.

Notably, the comparable performance of CFR and the Zn-free KM indicates that the mere presence of Zn is insufficient to ensure functional antibiofilm efficacy; rather, Zn bioavailability and release kinetics appear to be critical determinants.

Surface characteristics further influenced biofilm development. Although all glass ionomer materials exhibited relatively smooth surfaces at 2 h, CFR and KM developed more pronounced surface irregularities than CR by 4 h, accompanied by increased bacterial adhesion and clustering. These alterations are consistent with acid-mediated degradation of the surface matrix and dissolution of regions surrounding glass particles under biofilm challenge conditions [22].

In contrast, biofilms formed on the resin-based control (DS) appeared less mature than those on CFR and KM, potentially reflecting the release of residual monomers during polymerization, which have been reported to exert cytotoxic or growth-inhibitory effects on bacterial cells [23]. Surface cracks observed in all glass ionomer specimens are most likely attributable to dehydration artifacts associated with SEM vacuum preparation [24].

### 3.2. Glass Ionomer Composition, Zinc Incorporation, and Demineralization Resistance

Material-dependent differences were also evident in enamel and dentin demineralization. CR exhibited the greatest resistance to acid-induced mineral loss, whereas CFR and KM showed comparable performance, and DS demonstrated the poorest resistance. These findings indicate that material chemistry influences not only biofilm formation but also the extent of mineral dissolution at the restoration–tooth interface.

Zinc has been reported to promote dentin remineralization by preserving collagen cross-links [25,26] and enhancing calcium and phosphate accumulation within demineralized tissues [27]. In enamel, Zn may inhibit demineralization by binding to hydroxyapatite surfaces or precipitating as α-Zn_3_(PO_4_)_2_·4H_2_O, thereby limiting calcium and phosphate ion dissolution under acidic conditions [28,29].

Given the beneficial effects of zinc, the differential demineralization profiles observed among zinc-containing materials under biofilm derived acidic challenge suggest that the strategy by which zinc is incorporated into the material matrix constitutes a decisive determinant of material behavior.

In CFR, zinc is directly integrated into the glass phase as ZnO within a calcium-aluminum-zinc-fluoro-phosphosilicate network [30], whereas in CR, it is incorporated through BioUnion™ fillers composed of silicon dioxide (SiO_2_), zinc oxide (ZnO), calcium oxide (CaO), and fluoride (F) [6], residing as a discrete phase independent to the primary fluoro-aluminosilicate glass matrix (Table 1).

In conventional GICs, the acid-base setting reaction is initiated by polyacrylic acid attacking the glass network; cations leached from the glass surface subsequently crosslink the polyacrylic acid chains to form a polysalt matrix [31]. Distinct from conventional glass compositions, zinc within the glass network of CFR functions as a network modifier, disrupting Si–O–Si bridging bonds and rendering the glass structurally more reactive to acid challenge [32]. Although this structural characteristic accelerates the setting reaction, it may concurrently render the set material more susceptible to externally applied acid attack. In support of this hypothesis, Birant et al. reported that ChemFil Rock exhibited statistically significantly lower microhardness values following acidic aging compared to Equia Forte and Ketac Molar, attributing this outcome to hardness, interfiller particle gap size, morphology, and chemical composition of the material, and suggesting that the calcium-aluminum-zinc-fluorosilicate glass may be inherently less strong [33]. The irregular surface morphology observed in CFR following biofilm exposure (Figure 2f and Figure 3b) further corroborates the likelihood of acid driven wash-out and localized material dissolution. This interpretation may be further supported by recent atomistic investigations demonstrating that the structural integration and stability are governed by the interplay between atomic structure and interfacial configurations, which can directly influence resistance to structural degradation [34].

The incorporation of zinc as a nanoscale component within a discrete phase independent from the primary fluoro-alumino-silicate glass matrix in CR structurally shields the Si–O–Si bridging bonds of that matrix from ZnO-induced disruption, thereby allowing the matrix to retain its relative acid resistance, an effect that may account for the superior surface stability observed in CR (Figure 2e).

As reported by Naksagoon et al. and Kohno et al., Zn^2+^ release from BioUnion™ nanoparticles increases dramatically under acidic conditions, a finding that may be interpreted as indicative of selective and localized dissolution of the nanoparticulate phase rather than degradation of the primary matrix [35,36]. The incorporation of bioactive agents in nanoparticulate form has been associated with enhanced mechanical properties [37], augmented antibacterial efficacy [38], and reinforcement of dental hard tissues [39]. Considered within this framework, the nanoparticulate architecture of BioUnion™ may represent one of the principal structural determinants underlying the superior acid resistance and surface stability of CR observed in the present study.

While these properties collectively provide a coherent framework for explaining the differential demineralization profiles observed under acidic conditions, the surface degradation mechanisms of set zinc-reinforced glass ionomer cements remain incompletely elucidated. Further investigations encompassing atomistic modeling and advanced surface characterization techniques will substantially deepen the understanding of the long-term clinical durability of these materials.

Notably, the acid composition of CFR is reported inconsistently across the literature. Bahammam et al. [40] describe its liquid phase as “polyacrylic and tartaric acid,” whereas Zoergiebel & Ilie [30] report “polycarboxylic acid” for the same material. Since polycarboxylic acid, unlike polyacrylic acid, lacks the double bond necessary for chain-growth polymerization and subsequent cation crosslinking, this compositional ambiguity precludes definitive mechanistic conclusions regarding the crosslinking capacity of CFR’s acid system and its contribution to the structural integrity of the set cement. Future studies directly characterizing the liquid phase compositions of zinc-containing glass ionomer cements would substantially clarify the relationship between acid system chemistry and the mechanical and structural integrity of these materials.

### 3.3. Clinical Relevance

According to the findings of the present study zinc-containing GICs appear to exhibit enhanced antibiofilm activity compared to conventional GICs, with CR showing the greatest resistance to biofilm-induced mineral loss in both enamel and dentin. Considering these results, zinc-containing GICs may provide clinically relevant advantages in the management of high caries-risk patients, particularly in conditions associated with impaired plaque control or reduced salivary function. By limiting biofilm mediated demineralization and preserving mineral balance at the tooth-restoration interface, these materials may contribute to improved disease control and restoration longevity, supporting their use in minimally invasive approaches.

## 4. Materials and Methods

The restorative materials evaluated in this study included two zinc-containing glass ionomer cements (Zn-GICs): Caredyne Restore (CR) and ChemFil Rock (CFR), and a conventional zinc-free GIC (Ketac Molar, KM). For the assessment of biofilm-mediated demineralization, Dura Seal (DS), a resin-based material, was additionally included as a control. The properties and compositions of all materials are summarized in Table 1.

### 4.1. Evaluation of Biofilm Formation on the Surface of Materials

#### 4.1.1. Preparation of Specimens

Eighteen disk-shaped specimens (*n* = 6 per material; 6 mm in diameter and 2 mm in thickness) were prepared using silicone molds in accordance with the manufacturers’ instructions. Caredyne Restore (powder/liquid ratio: 2.3 g/1.0 g) and Ketac Molar (powder/liquid ratio: 12.5 g/8.5 mL) were manually mixed according to the manufacturers’ recommended powder to liqid ratios. ChemFil Rock, supplied in capsule form, was mixed using a capsule mixer at 4000 rpm for 15 s.

To obtain flat and void-free surfaces, the materials were pressed between a transparent polyester strip and a glass slide. After 6 min, the strip was removed. All specimens were stored at 37 °C and 100% relative humidity for 24 h.

#### 4.1.2. Preparation of Bacterial Suspensions

*S. mutans* MT 8148, *S. sobrinus* 6715, *S. gordonii* ATCC 10558, *S. mitis* ATCC 6249 and *L. casei* ATCC 393 strains were used in this study. All strains were precultured individually at 37 °C; streptococci in Brain Heart Infusion (BHI) broth (BD Bacto™, Becton, Dickinson and Company, Sparks, MD, USA) and Lactobacillus strains in MRS broth (BD Difco™ Becton, Dickinson and Company Sparks, MD, USA). Subsequently, all strains were combined to co-culture for 18 h in a mixture of BHI and MRS broth at 37 °C. All cultivations were carried out using AnaeroPouch of Mitsubishi™ AnaeroPack-CO_2_ (Mitsubishi Gas Chemical Company, Tokyo, Japan). The bacterial cells were washed with phosphate-buffered saline (PBS) (FUJIFILM Wako Pure Chemical Corporation, Osaka, Japan) and suspended in PBS to an optical density (OD_490_) of approximately 0.5. The prepared bacterial suspension was transferred to the oral biofilm reactor.

#### 4.1.3. Biofilm Formation in an Oral Biofilm Reactor on the Material Surface

Biofilms were cultivated on the material surfaces within two identical water jacketed oral biofilm reactor chambers. The specimens were positioned horizontally in a Teflon holder using utility wax (GC Corporation, Tokyo, Japan) and inserted into the lower openings of the chambers with silicone stoppers. Each chamber was sealed with a silicone stopper fitted with five 21-gauge stainless steel tubes. The system was maintained at 37 °C during incubation. The ends of the tubes were connected to silicone tubing attached to peristaltic pumps, which were controlled via a computer-based system (EYELA EPC-2000, Tokyo Rika, Tokyo, Japan).

**Figure 6:**
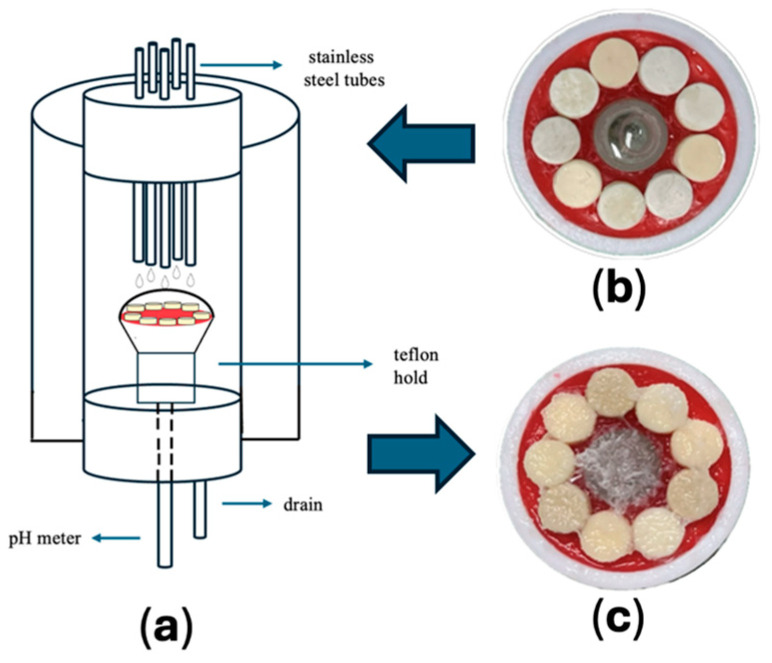
Schematic representation of the oral biofilm reactor (**a**). Specimens positioned in the Teflon holder before biofilm formation (**b**) Specimens in the Teflon holder after 12 h of multispecies biofilm development (**c**).

One tube was allocated for delivery of the bacterial suspension, two for HI-sucrose medium, and the remaining two for PBS. Each liquid was supplied to the chambers at a constant flow rate of 6 mL/h, allowing continuous delivery onto the center of the specimen holder. The falling droplets formed liquid domes that spread across the specimen surfaces through gravitational mixing upon impact [41,42]. Biofilms were allowed to form on the material surfaces for a period of 12 h (Figure 6).

#### 4.1.4. Quantitative Assessment of Biofilms

After completion of the experimental procedures, biofilm on each specimen was quantified by separately measuring bacterial cell density and water-insoluble glucan (WIG) content. Non-adherent bacteria were removed by immersing the Teflon holders in PBS, and each specimen with its surface-associated biofilm was transferred to an individual Eppendorf tube. Biofilm detachment was achieved by adding 1 mL of 30% acetone (FUJIFILM Wako Pure Chemical Corporation, Osaka, Japan), followed by removal of the biofilm-free specimens. The remaining suspension was centrifuged at 10,000× *g* for 5 min to eliminate residual acetone.

To separate bacterial cells from the WIG matrix, 0.5 mol/L sodium hydroxide (NaOH) (FUJIFILM Wako Pure Chemical Corporation, Osaka, Japan) solution was added to each tube to disrupt the triple-helix structure of glucan. Following centrifugation at 10,000× *g* for 5 min, the WIG-containing supernatant was carefully transferred to new microtubes, while the bacterial pellets were resuspended in 1 mL PBS. 100 µL of each suspension was transferred in triplicate to 96-well plates. Bacterial quantification was performed by turbidimetric analysis at 490 nm using a spectrophotometer (Microplate Reader Model 680, Bio-Rad, Hercules, CA, USA). Absorbance values were normalized to the surface area of each specimen and expressed as optical density per square millimeter (OD/mm^2^).

The NaOH-containing supernatants recovered after centrifugation were transferred to 10 mL glass tubes for WIG quantification. For the colorimetric assay, 250 µL of each WIG sample was added to 500 µL of 5% phenol solution and mixed thoroughly. Subsequently, 1 mL of concentrated H_2_SO_4_ (95.0%, FUJIFILM Wako Pure Chemical Corporation, Osaka, Japan) was rapidly delivered into each phenol-WIG mixture to initiate an exothermic reaction. The resulting solution was allowed to cool for 30 min, after which 200 µL of each sample was transferred in triplicate to 96-well flat-bottom plates, and absorbance was measured at 490 nm using the same microplate reader. WIG concentrations were determined by reference to a standard curve constructed with pure glucose (FUJIFILM Wako Pure Chemical Corporation, Osaka, Japan), and results were expressed as µg/mL/mm^2^ [10].

### 4.2. Evaluation of Early Biofilm Formation on Material Surfaces

Early-stage biofilms were formed on the material surfaces for 2 h and 4 h according to the protocol described in Section 2.1. The specimens were immediately chemically fixed in 4% paraformaldehyde and 2.5% glutaraldehyde, followed by washing and dehydration. Surface morphology was then examined using scanning electron microscopy (SEM).

### 4.3. Evaluation of Enamel and Dentin Demineralization Induced by Biofilm-Mediated Acid Exposure

This study was approved by the Ethics Committee of Ege University Medical Research Ethics Committee (Approval No: 24-2T/11). All specimens were anonymized to ensure confidentiality and were handled in accordance with the ethical standards set forth in the Declaration of Helsinki.

#### 4.3.1. Preparation of Specimens

**Figure 7:**
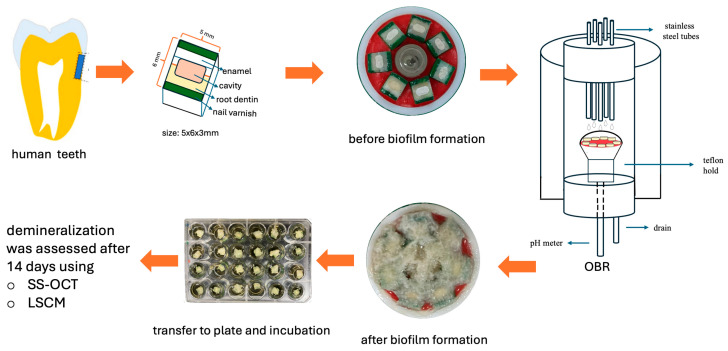
Stepwise schematic illustrating the preparation of enamel-dentin specimens with restored cavities, transfer of specimens to the oral biofilm reactor (pre-biofilm stage), multispecies biofilm development, subsequent transfer to multi-well plates for incubation, and post-challenge imaging by SS-OCT and LSCM after 14 days. Arrows indicate the chronological sequence of the experimental procedures. SS-OCT: swept-source optical coherence tomography; LSCM: laser scanning confocal microscopy.

A total of 24 specimens (6 per group) (5 mm × 6 mm × 3 mm), each comprising both enamel and dentin tissues, were prepared from extracted human teeth using a precision saw (Isomet 1000, Buehler, Lake Bluff, IL, USA). The specimens were sequentially polished under running water with silicon carbide (SiC) abrasive papers of #800, #1000, #1500, and #2000 grit. Standardized cavities (4 × 2 × 2 mm) were prepared using a diamond bur, followed by ultrasonic cleaning for 10 min. Restorative materials were prepared according to the manufacturers’ instructions and placed into the cavities. To prevent peripheral acid exposure, 1 mm-wide zone of enamel and dentin along the peripheral margins of the 5 mm occlusal surface was coated with nail varnish (Revlon Inc., New York, NY, USA) (Figure 7).

#### 4.3.2. Growth of Biofilms on the Specimens

The specimens were mounted horizontally on a Teflon holder within the oral biofilm reactor (OBR) using utility wax (GC, Tokyo, Japan). As described in Section 4.1.2, the same bacterial species and culture media required for biofilm formation were prepared. Biofilms were allowed to develop on the specimen surfaces for 24 h. Subsequently, each specimen with the associated biofilm was transferred to a 24-well microplate, with one specimen per well. One milliliter of HI-sucrose medium was added to each well, and the plates were incubated at 37 °C. The medium was refreshed every 2 days over a 14-day period. At the end of the incubation period, the specimens were rinsed with PBS, and the biofilm was removed using 30% acetone.

#### 4.3.3. Swept-Source Optical Coherence Tomography (SS-OCT)

Swept-source optical coherence tomography (SS-OCT), a non-destructive imaging technique capable of assessing tissue microstructure, was employed for evaluation. The system operated over a wavelength range of 1260–1360 nm, centered at 1310 nm, with a sweep rate of 20 kHz [43]. The axial and lateral resolutions were 12.0 μm and 17.0 μm, respectively. Raw SS-OCT data were analyzed using ImageJ software (version 1.54g, National Institutes of Health, Bethesda, MD, USA).

#### 4.3.4. Laser Scanning Confocal Microscope (LSCM)

Following SS-OCT imaging, all specimens were embedded in epoxy resin (EpoxiCure; Buehler, Lake Bluff, IL, USA). The embedded specimens were sectioned using a low-speed diamond saw (Isomet; Buehler, Lake Bluff, IL, USA) and subsequently polished under running water with #1000-, #1500-, and #2000-grit silicon carbide papers, yielding slices approximately 1.0 ± 0.2 mm in thickness. The sections were examined using laser scanning confocal microscopy (LSCM; 1LM21H/W, Lasertec Co., Yokohama, Japan). Lesion depth was quantified using ImageJ software (National Institutes of Health, Bethesda, MD, USA). Schematic illustrations were prepared using Microsoft PowerPoint (Microsoft Corp., Redmond, WA, USA).

#### 4.3.5. Quantitative Analysis of Enamel and Dentin Demineralization

In the SS-OCT and LSCM images, the intact outer surfaces of enamel and dentin that were protected from acid exposure served as reference points. The lesion onset was defined at the point where the reference line intersected the enamel or dentin at a distance of 200 µm from the material interface. Lesion depths were subsequently quantified from this defined point.

### 4.4. Statistical Analysis

Data were assessed for normality using the Shapiro–Wilk test and for homogeneity of variances using Levene’s test. Bacterial counts (bacteria/mm^2^), water-insoluble glucan levels (WIG/mm^2^), and demineralization depth were analyzed using one-way ANOVA, followed by Tukey’s honestly significant difference (HSD) post hoc test for multiple comparisons at a 95% confidence level. Statistical analyses were conducted using the Python libraries statsmodels (version 0.14.0), pandas (version 2.0.3), and NumPy (version 1.24.3).

## 5. Conclusions

Within the limitations of this in vitro study, CR, a Zn-GIC, demonstrated superior antibiofilm performance and the greatest resistance to biofilm mediated enamel and dentin demineralization among all tested materials. In contrast, CFR, despite containing zinc, performed comparably to the conventional zinc-free GIC, highlighting that the efficacy of Zn-GICs depends on the mode of zinc incorporation rather than its mere presence.

The strengths of this study include the use of a clinically relevant multi-species biofilm model and the integration of complementary imaging techniques to assess both biofilm formation and biofilm mediated mineral loss. These findings, however, should be interpreted with caution, as in vitro conditions cannot fully replicate the biological complexity of the oral environment, including the dynamic interplay of salivary proteins, immune factors, and occlusal forces. Future well designed randomized clinical trials, particularly those enrolling patient populations with varying salivary flow rates and dietary profiles, are warranted to validate the clinical relevance and long-term preventive potential of Zn-GIC.

## Figures and Tables

**Table 1:** Composition and properties of the experimental materials.

Material	Manufacturer/ Country	Material Composition	Lot Number
Caredyne Restore (CR) (Zn-GIC)	GC Corporation Tokyo, Japan	Powder: fluoro-zinc silicate glass, fluoro-alumino silicate glass Liquid: polyacrylic acid, acrylic acid and tri-carboxylic acid copolymer, water	Powder: 242408011 Liquid: 2408011
ChemFil Rock (CFR) (Zn-GIC)	Dentsply Corporation Konstanz, Germany	Capsule: polycarboxylic acid, titanium dioxide pigments, calcium-aluminum-zinc-fluoro-phosphor-silicate glass, iron oxide pigments, tartaric acid, water	2204000525
Ketac Molar Easymix (KM) (zinc-free GIC)	3M ESPE Corporation Seefeld, Germany	Powder: 5% copolymer acid (acrylic and maleic acid), Al-Ca-La fluoro-silicate glass Liquid: Tartaric acid, polyalkenoic acid, water	Powder: 10445004 Liquid: 10383576
Dura Seal (DS) (Resin)	Reliance Dental, Alsip, IL, USA	self-curing resin	Powder: 050819 Liquid: 022420

## Data Availability

The original contributions presented in this study are included in the article. Further inquiries can be directed to the corresponding author.
